# Factors associated with habitual time spent in different physical activity intensities using multiday accelerometry

**DOI:** 10.1038/s41598-020-57648-w

**Published:** 2020-01-21

**Authors:** Lina Jaeschke, Astrid Steinbrecher, Heiner Boeing, Sylvia Gastell, Wolfgang Ahrens, Klaus Berger, Hermann Brenner, Nina Ebert, Beate Fischer, Karin Halina Greiser, Wolfgang Hoffmann, Karl-Heinz Jöckel, Rudolf Kaaks, Thomas Keil, Yvonne Kemmling, Alexander Kluttig, Lilian Krist, Michael Leitzmann, Wolfgang Lieb, Jakob Linseisen, Markus Löffler, Karin B. Michels, Nadia Obi, Annette Peters, Sabine Schipf, Börge Schmidt, Melanie Zinkhan, Tobias Pischon

**Affiliations:** 1 Molecular Epidemiology Group, Max Delbrück Center for Molecular Medicine in the Helmholtz Association (MDC), Berlin, Germany; 2 Division of Epidemiology, German Institute of Human Nutrition Potsdam-Rehbruecke, Potsdam-Rehbruecke, Germany; 3 Leibniz Institute for Prevention Research and Epidemiology – BIPS, Bremen, Germany; 4 Institute of Statistics, Faculty of Mathematics and Computer Science, University of Bremen, Bremen, Germany; 5 Institute of Epidemiology and Social Medicine, University of Münster, Münster, Germany; 6 Division of Clinical Epidemiology and Aging Research, German Cancer Research Center, INF 581 Heidelberg, Germany; 7 German Diabetes Center (DDZ), Leibniz Center for Diabetes Research, Heinrich Heine University, Institute for Biometrics and Epidemiology, Düsseldorf, Germany; 8 Department of Epidemiology and Preventive Medicine, University of Regensburg, Regensburg, Germany; 9 German Cancer Research Center (DKFZ), Heidelberg, Germany; 10 Section Epidemiology of Health Care and Community Health, Institute for Community Medicine, University Medicine Greifswald, Greifswald, Germany; 11 Institute for Medical Informatics, Biometry and Epidemiology (IMIBE), University Hospital Essen, University of Duisburg-Essen, Essen, Germany; 12 Institute for Social Medicine, Epidemiology and Health Economics, Charité – Universitätsmedizin Berlin, Berlin, Germany; 13 Department of Epidemiology, Helmholtz-Centre for Infection Research, Braunschweig, Germany; 14 Institute of Medical Epidemiology, Biometry and Informatics, Martin-Luther-University, Halle (Saale), Germany; 15 Institute of Epidemiology, Kiel University, Kiel, Germany; 16 Chair of Epidemiology, LMU Munich at UNIKA-T, Augsburg, Germany; 17 Helmholtz Zentrum München, IRG Clinical Epidemiology, Neuherberg, Germany; 18 Institute for Medical Informatics, Statistics and Epidemiology, Leipzig, Germany; 19 Institute for Prevention and Cancer Epidemiology, Faculty of Medicine and Medical Center, University of Freiburg, Freiburg, Germany; 20 Institute for Medical Biometry and Epidemiology, University Medical Center Hamburg-Eppendorf, Hamburg, Germany; 21 Institute of Epidemiology, Helmholtz Zentrum München - German Center for Health and Environment, Neuherberg, Germany; 22 Institute for Community Medicine, University Medicine Greifswald, Greifswald, Germany; 23 Charité – Universitätsmedizin Berlin, Berlin, Germany; 24 German Center for Cardiovascular Research (DZHK), partner site Berlin, Berlin, Germany; 25 MDC/BIH Biobank, Max Delbrück Center for Molecular Medicine and Berlin Institute of Health, Berlin, Germany

**Keywords:** Epidemiology, Epidemiology, Risk factors

## Abstract

To investigate factors associated with time in physical activity intensities, we assessed physical activity of 249 men and women (mean age 51.3 years) by 7-day 24h-accelerometry (ActiGraph GT3X+). Triaxial vector magnitude counts/minute were extracted to determine time in inactivity, in low-intensity, moderate, and vigorous-to-very-vigorous activity. Cross-sectional associations with sex, age, body mass index, waist circumference, smoking, alcohol consumption, education, employment, income, marital status, diabetes, and dyslipidaemia were investigated in multivariable regression analyses. Higher age was associated with more time in low-intensity (mean difference, 7.3 min/d per 5 years; 95% confidence interval 2.0,12.7) and less time in vigorous-to-very-vigorous activity (−0.8 min/d; −1.4, −0.2), while higher BMI was related to less time in low-intensity activity (−3.7 min/d; −6.3, −1.2). Current versus never smoking was associated with more time in low-intensity (29.2 min/d; 7.5, 50.9) and less time in vigorous-to-very-vigorous activity (−3.9 min/d; −6.3, −1.5). Finally, having versus not having a university entrance qualification and being not versus full time employed were associated with more inactivity time (35.9 min/d; 13.0, 58.8, and 66.2 min/d; 34.7, 97.7, respectively) and less time in low-intensity activity (−31.7 min/d; −49.9, −13.4, and −50.7; −76.6, −24.8, respectively). The assessed factors show distinct associations with activity intensities, providing targets for public health measures aiming to increase activity.

## Introduction

Habitual physical activity (PA) is crucial in the aetiology of noncommunicable diseases^1^. Insufficient PA is one of the leading risk factors for mortality and disability-adjusted life years globally^1,2^. For adults, the World Health Organization (WHO) recommends at least 150 minutes of moderate or 75 minutes of vigorous PA per week or metabolic equivalents^1^. Previous studies have shown that meeting these criteria reduces morbidity and mortality risk^3,4^.

Knowing factors associated with PA is important, since modifiable factors might represent targets for public health measures that aim to increase PA, while unmodifiable factors might allow identification of physically active and inactive target subgroups^5^. Sex, age, anthropometry, health, smoking, alcohol consumption, marital status, education, employment, and income are important factors from different domains that may be related to PA, including biology, behaviour, socio-culture, and socio-economy. However, evidence on a relationship to PA is inconsistent for most of these factors and mainly based on self-reported PA^6–13^, being prone to measurement error, especially regarding PA intensities^14^. Assessing both PA duration and intensity is important in epidemiologic studies, as both are independent behaviours and risk factors^3,15^. Accelerometers detect accelerations of the body under free-living conditions, capturing PA duration and intensity more precisely than self-reports. However, previous studies largely rely on uniaxial accelerometry applied during waking only^16–19^, while new cohorts, like the German National Cohort (NAKO Gesundheitsstudie) or the UK Biobank use triaxial 24h-accelerometry^20–22^. Further, while convincing or probable evidence from systematic analyses on PA-related factors in general is scarce^6–11,13,23,24^, to our knowledge, it is lacking for 24h-accelerometry-based PA intensities in the adult German population.

The aim of this study was to investigate factors associated with habitual time spent in different activity intensities, and to estimate the likelihood of meeting the WHO PA recommendation in a sample from the general German adult population using multiday 24h-accelerometry.

## Materials and Methods

### Pretest of the German National Cohort (NAKO Gesundheitsstudie)

Data were collected as part of a pretest of the German National Cohort (NAKO Gesundheitsstudie)^20^. The NAKO is an ongoing population-based cohort study that recruited 205,000 adults aged 20–69 years from the general population in 18 study centres distributed across Germany, in order to study causes and mechanisms of major chronic diseases^20,21^. Pretests were conducted upfront with the primary aim to test and implement infrastructures and study protocols^25^. Data used in the present analyses were collected during pretest 2 between May 2012 and April 2013. Each study centre was expected to draw an age- and sex-stratified random sample from local municipal population registries (50% men, 10-years age groups from 20–69 years, with 10% of participants in each of the two younger age groups, and 26.6% of participants in each of the other age groups) to examine at least 100 participants according to a common protocol^20,25^. Inclusion criteria were residence in the catchment area of the municipal registries covered by the respective study centre, age 20–69 years, proficiency of the German language, and ability to provide informed consent.

The study protocol of the NAKO pretest was approved by the ethics committee of the Bayerische Landesärztekammer (study center: Augsburg), the Charité – Universitätsmedizin Berlin (Berlin-Center, Berlin-North, Berlin-South/Brandenburg), the Ärztekammer Niedersachsen (Hannover/Braunschweig), the Ärztekammer Bremen (Bremen), the Medical Faculty of the Heinrich-Heine-Universität Düsseldorf (Düsseldorf), the Albert-Ludwigs-Universität Freiburg (Freiburg), the Medical Faculty of the Martin-Luther-Universität Halle-Wittenberg (Halle), the Ärztekammer Hamburg (Hamburg), the Medical Faculty Heidelberg (Heidelberg), the Medical Faculty of the Christian-Albrechts-Universität Kiel (Kiel), the Ärztekammer Westfalen-Lippe and the Medical Faculty of the Westfälische Wilhelms-Universität (Münster), the Universitätsmedizin Greifswald (Neubrandenburg), the Medical Faculty of the Universität Regensburg (Regensburg), and the Ärztekammer des Saarlandes (Saarbrücken), as well as by the local data protection officers. All investigations were carried out in accordance with the relevant guidelines and regulations, and written informed consent was obtained from all participants before inclusion^20^.

### Data collection

Data collection followed a standardized protocol across study centres. Participants visited the study centre and performed a personal computer-assisted interview on age, smoking status, alcohol consumption, highest level of education, employment status, net household income, marital status, and history of diabetes mellitus and dyslipidaemia^20,21^. Participants underwent an anthropometric measurement by trained personnel according to WHO guidelines^21^. Body height (cm) was measured using the stadiometer SECA 285 (Hamburg, Germany) or an equivalent model, body weight (kg) using the bioelectrical impedance analysis device SECA mBCA 515 (Hamburg, Germany) or an equivalent model, and waist circumference (WC, cm) using the measuring tape SECA 201 (Hamburg, Germany). Body mass index (BMI) was calculated as body weight divided by the square of body height (kg/m²). All measurements were performed with an accuracy of one decimal place^21^.

PA was assessed by the triaxial accelerometer GT3X+ (ActiGraph LLC, Pensacola, FL, USA). Participants were asked to continuously wear the accelerometer 24 h/d on the right hip for one week and to send it back by mail thereafter. They were instructed to take off the device for high contact sports, sauna sessions, and water-based activities >30 minutes only^21^. ActiLife (ActiGraph LLC, Pensacola, FL, USA) software was used to process the acceleration data (initialization and downloading: versions 6.4.3/5, 6.5.2/3, and 6.7.3; data processing: versions 6.12.1 to 6.13.2). Raw triaxial acceleration data were sampled by a 12 bit analogue-to-digital converter (dynamic range, ±6 G) using a constant 100 Hz sampling rate (stored at 100 Hz rate), with the filter set to ‘normal’ (default) and the ‘Idle Sleep Mode’ being disabled. Raw triaxial acceleration data were converted into 10-seconds epochs on site and were transferred to a central data management facility. Each study centre was supposed to apply 24h-accelerometry in at least 20 participants.

### Study sample

**Figure 1:**
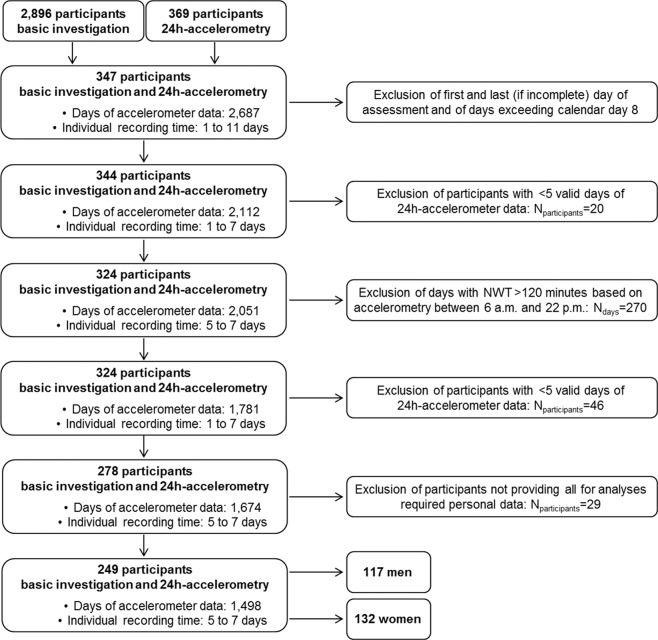
Selection of the study population from pretest 2 of the German National Cohort (NAKO Gesundheitsstudie). Initially, in pretest 2 of the German National Cohort (NAKO Gesundheitsstudie), 2,896 participants provided data on basic characteristics (interview and anthropometry), 369 participants provided data on 24h-accelerometry (GT3X+, ActiGraph LLC, Pensacola, FL, USA), and 347 participants provided data on both. Finally, 249 participants fulfilled all inclusion criteria and were included for the present analyses on factors potentially associated with 24h-accelerometry-based physical activity intensities. NWT, non-wear time.

In total 2,896 participants were included in pretest 2 (response, 18.7%). Of these, 347 took part in 24h-accelerometry (Fig. 1). From these, we excluded the first day of assessment (study centre visit) and all days exceeding day 8. Finally, the last day of assessment was excluded when incomplete. For final inclusion, a data set needed to encompass ≥5 days not showing any accelerometer non-wear time >120 minutes during a presumed waking period between 6 AM and 10 PM automatically detected by ActiLife. These decisions were based on our previous studies, showing (1) that having ≥5 days of 24h-accelerometry data available provides sufficient validity for PA assessment (expected correlation between observed and true PA parameter ≥0.85) and (2) that automated non-wear time detection is most specific and sensitive when using a 120-min algorithm^26,27^. We further excluded participants with missing information on any variables included in the multivariable model. In total, 98 participants were excluded, resulting in 249 participants (117 men, 132 women) being finally included in the present analyses.

### Operationalization of physical activity intensities

For each participant and day, the triaxial ‘vector magnitude counts per minute’ (cpm) was used to determine PA parameters based on the triaxial-derived algorithm ‘Freedson Adult VM3 (2011)’ implemented in ActiLife. Referring to metabolic equivalents of tasks (METs), this algorithm classifies 0–2,690 cpm as light (equivalent to <3.0 METs; e.g., slow walking), 2,691–6,166 cpm as moderate (3.0–5.99 METs; e.g., gymnastics), 6,167–9,642 cpm as vigorous (6.0–8.99 METs; e.g., jogging), and ≥9,643 cpm as very vigorous activity (≥9.0 METs; e.g., carrying loads upstairs, squash)^28^. As reported previously, we further subdivided ‘light’ activity into time in ‘inactivity’ (0–78 cpm) and in ‘low-intensity’ activity (79–2,690 cpm)^27^. Since mean daily time in very vigorous activity was short (geometric mean, GM, 0.16 min/d), we summed it with vigorous activity to vigorous-to-very-vigorous (VV) activity. Times spent in activity intensities were determined as mean min/d.

Additionally, we characterized participants according to WHO PA recommendations, which requires time in moderate or vigorous activity to be spent in bouts of ≥10 minutes^1^. For each participant, we determined the total time spent in 10-minutes bouts in moderate or VV activity, divided the sum by the number of assessment days, and multiplied by 7 to obtain mean week estimates. Estimates ≥150 minutes of moderate or ≥75 minutes of VV activity per week were classified as ‘meeting WHO recommendation’. Otherwise, we determined if participants achieved a ‘metabolic equivalent’ of these, as mentioned, although not defined, by the WHO^1^. Fulfilling the WHO criteria is equivalent to achieving 450 METs/week, when using 3 and 6 METs as limits for moderate and vigorous activity, respectively^1^. To acknowledge higher activity intensities within both intensity levels, we multiplied the weekly bouts estimates in moderate and VV activity by 4 and 8 METs, respectively^29^. If participants achieved ≥450 METs/week based on moderate and VV activity, this was classified as ‘meeting WHO recommendation’. Participants not meeting any of the aforementioned criteria were classified as ‘not meeting WHO recommendation’.

### Statistical analyses

To investigate factors associated with time spent in inactivity and in low-intensity, moderate, and VV activity, we used four separate linear regression models with robust variance estimation^30^, each of the PA measures as single outcome, and the following independent variables: sex (male, female), age (continuously), BMI (continuously), WC (continuously, residually adjusted for BMI), smoking status (never, current, or former), alcohol consumption (never, ≤1×/month, 2–4×/month, 2–3×/week, or ≥4×/week), university entrance qualification (yes, no), employment status (full time, part time or not employed (including retired)), net household income/month (<2,500€, 2,500–3,999€, ≥4,000€, or not available), marital status (married, not married), diabetes mellitus (yes, no), dyslipidaemia (yes, no), and study centre (dummy variables). We tested for sex differences by further including interaction terms with sex for each independent variable, and we performed analyses stratified by sex.

To investigate factors related to meet the WHO PA recommendation (yes, no), we used logistic regression analyses and the same respective models as conducted for the linear analyses.

In sensitivity analyses, all analyses were repeated using a sample that was selected by using a more conservative 60-minutes instead of 120-minutes non-wear time algorithm, resulting in 201 participants (86 men, 115 women) and a total of 1,070 24h-accelerometry days. In a further analysis, we determined PA intensities using common uniaxial-derived cut points proposed by Freedson *et al*., defining 0–99 cpm measured by the vertical axis as ‘sedentary’, 100–1,951 cpm as ‘light’, 1,952–5724 cpm as ‘moderate’, and ≥5,725 cpm as VV PA^31^ and repeated linear analyses. Finally, since 76% of persons in the group ‘not employed’ were retired, we repeated linear analyses by replacing ‘employment status’ by ‘retired, yes vs. no’ and ‘not employed, yes vs. no’ (yes: including jobless persons, persons permanently unable to work, and housewives/househusbands).

To test for differences between included and excluded participants, unpaired t-tests or Mann-Whitney U tests, respectively, were used for continuous variables, and Chi-Square tests were performed for discrete variables.

P-values presented are two-tailed, with p < 0.05 considered statistically significant. To account for multiple testing using Bonferroni adjustments, p-values < 0.01 in the linear and logistic regression analyses (four and one regression models, respectively) and <0.0009 in the sex difference analyses (11 interaction terms included in each of the five regression models) were considered statistically significant. Analyses were performed using SAS® Enterprise Guide® (version 7.1; SAS Institute Inc., Cary, NC).

## Results

In total, 249 participants (47.0% men) were included (Table 1, and Supplementary Table S1). Compared to participants excluded from analyses (n = 98), participants included were more likely to have a net household income >4,000€/month (23.7% versus 10.2%) and higher WC (90.6 cm versus 87.4 cm); otherwise, basic characteristics were similar (data not shown).

**Table 1:** Basic characteristics of study participants, 2012–2013.

	Total (N = 249)		Men (n = 117)		Women (n = 132)	
	**mean**	**SD**	**mean**	**SD**	**mean**	**SD**
age, years	51.3	10.8	50.9	11.1	51.6	10.7
height, cm	171.0	9.5	178.2	6.8	164.6	6.6
weight, kg	77.2	14.5	86.7	12.3	68.8	10.5
BMI, kg/m²	26.3	4.0	27.4	3.9	25.4	3.8
WC, cm	90.6	12.9	98.3	11.8	83.8	9.7
	**n**	**%**	**n**	**%**	**n**	**%**
smoking status						
never	101	40.6	44	37.6	57	43.2
current	58	23.3	26	22.2	32	24.2
former	90	36.1	47	40.2	43	32.6
alcohol consumption						
never	7	2.8	3	2.6	4	3.0
max. 1×/month	56	22.5	15	12.8	41	31.1
2–4×/month	74	29.7	31	26.5	43	32.6
2–3×/week	64	25.7	34	29.1	30	22.7
≥4×/week	48	19.3	34	29.1	14	10.6
school education						
university entrance qualification	120	48.2	65	55.6	55	41.7
no university entrance qualification	129	51.8	52	44.4	77	58.3
employment status						
full time	135	54.2	85	72.6	50	37.9
part-time	62	24.9	11	9.4	51	38.6
not employed	52	20.9	21	17.9	31	23.5
net household income per month						
<2,500€	86	34.5	41	35.0	45	34.1
2,500–4,000€	92	36.9	40	34.2	52	39.4
>4,000€	59	23.7	33	28.2	26	19.7
n. a.	12	4.8	3	2.6	9	6.8
marital status						
married	156	62.7	79	67.5	77	58.3
not married	93	37.3	38	32.5	55	41.7
diabetes mellitus	18	7.2	11	9.4	7	5.3
dyslipidaemia	74	29.7	41	35.0	33	25.0

Information was derived from self-reports during a personal interview, anthropometric measures were taken by trained personnel.

BMI, body mass index; n, number; n. a., not available; SD, standard deviation; WC, waist circumference.

Most participants provided 24h-accelerometry data for 6 observation days (53.4%; 5 days: 22.5%; 7 days: 24.1%; Table 2). Men spent more time in inactivity and less time in low-intensity activity than women (p = 0.02 and p = 0.002, respectively). About one third of participants met the WHO PA recommendation, with 13.3% and 7.6% fulfilling the criteria of 150 min/week moderate and 75 min/week VV activity, respectively, spent in bouts of at least 10 minutes, and 11.2% meeting the metabolic equivalent. Men were slightly less likely to meet the criteria than women (p = 0.04).

**Table 2:** Physical activity parameters of study participants*.

	Total (N = 249)		Men (n = 117)		Women (n = 132)	
**PA parameter**	**GM**	**95% CI**	**GM**	**95% CI**	**GM**	**95% CI**
time in inactivity, min/d	1010.6	(999.6, 1021.8)	1024.3	(1008.1, 1040.8)	998.7	(983.8, 1013.8)
time in low-intensity activity, min/d	327.0	(318.4, 335.9)	312.8	(301.0, 325.0)	340.2	(328.1, 352.7)
time in moderate activity, min/d	78.8	(75.0, 82.9)	79.5	(73.8, 85.5)	78.3	(73.0, 83.9)
time in VV activity, min/d	2.7	(2.3, 3.2)	3.0	(2.4, 3.8)	2.5	(2.00, 3.1)
**10-minutes bouts of activity**	**median**	**IQR**	**median**	**IQR**	**median**	**IQR**
time in bouts of moderate activity, min/week	44.8	(12.3, 100.0)	35.0	(12.3, 78.2)	51.9	(12.4, 120.7)
time in bouts of VV activity, min/week	0.0	(0.0, 0.0)	0.0	(0.0, 0.0)	0.0	(0.0, 0.0)
**meeting WHO PA recommendation**^**a**^	**n**	**%**	**n**	**%**	**n**	**%**
no	169	67.9	87	74.4	82	62.1
yes^c^	80	32.1	30	25.6	50	37.9
based on moderate activity	33	13.3	13	11.1	20	15.2
based on VV activity	19	7.6	8	6.8	11	8.3
based on metabolic equivalent	28	11.2	9	7.7	19	14.4

95% CI, 95% confidence interval; cpm, counts per minute; GM, geometric mean; IQR, interquartile range; min/d, minutes per day; n, number; PA, physical activity; VV, vigorous-to-very-vigorous; WHO, World Health Organization.

*Activity intensities were determined based on triaxial 24h-accelerometry vector magnitude defining 0–78 cpm as ‘inactivity’, 79–2,690 cpm as ‘low-intensity’, 2,691–6,166 cpm as ‘moderate’, and ≥6,167 cpm as VV activity^27,28^.

^a^‘Meeting WHO PA recommendation’ (‘yes’) was defined as accumulating ≥150 min/week or ≥75 min/week of vigorous activity/week (here: VV activity) (mean weekly estimates: mean min/d per participant multiplied by 7), spent in activity bouts ≥10 minutes, or an equivalent combination of these^1^. For the latter metabolic equivalents of tasks (METs)/week were calculated, when multiplying mean weekly estimates in moderate and VV activity by 4 and 8 METs, respectively, as described before^29^. Achieving with the sum of both ≥450 METs/week, this was classified as ‘meeting WHO PA recommendation’. Not meeting any of the aforementioned criteria was classified as ‘not meeting WHO recommendation’.

**Bold**: *p*-value < 0.05.

### Linear regression analyses

Regarding the multivariable-adjusted associations of investigated factors with time spent in different PA intensities, higher age was significantly associated with more time spent in low-intensity (7.3 min/d per 5 years; 95% confidence interval, CI, 2.0 to 12.7) but less time in VV activity (−0.8 min/d; −1.4 to −0.2) (Table 3). Furthermore, a 1-unit higher BMI was significantly associated with less time in low-intensity activity (−3.7 min/d, −6.3 to −1.2). Additionally, current as compared to never smoking was significantly associated with more time in low-intensity (29.2 min/d; 7.5 to 50.9) but less time in VV activity (−3.9 min/d; −6.3 to −1.5). Having a university entrance qualification as compared to no such qualification was significantly associated with more inactivity time (35.9 min/d; 13.0 to 58.8) and less time spent in low-intensity activity (−31.7 min/d; −49.9 to −13.4). Finally, not versus full time employed persons spent more time in inactivity (66.2 min/d; 34.7 to 97.7) but less time in low-intensity activity (−50.7 min/d; −76.6 to −24.8). When adjusted for multiple testing, sex differences in the multivariable-adjusted associations of investigated factors with time in activity intensities were not statistically significant (Supplementary Table S2).

**Table 3:** Multivariable association of physical activity-related factors and time in different activity intensities^*^, total (N = 249).

Potential factors	time in inactivity, min/d			time in low-intensity activity, min/d			time in moderate activity, min/d			time in VV activity, min/d		
	β	95% CI	p	β	95% CI	p	β	95% CI	p	β	95% CI	p
sex (men vs. women)	9.1	(−19.6, 37.9)	0.53	−19.3	(−41.8, 3.2)	0.09	9.2	(−4.2, 22.6)	0.18	1.0	(−3.3, 5.3)	0.65
age (5 years)	−6.1	(−13.0, 0.7)	0.08	**7.3**	**(2.0, 12.7)**	**0.007**	−0.4	(−2.8, 2.1)	0.77	**−0.8**	**(−1.4, −0.2)**	**0.007**
BMI, kg/m²	3.9	(0.6, 7.3)	0.02	−**3.7**	**(**−**6.3**, −**1.2)**	**0.005**	0.1	(−1.4, 1.5)	0.93	−0.3	(−0.6, 0.1)	0.12
WC, cm^a^	1.6	(−0.3, 3.5)	0.10	−0.5	(−2.0, 1.0)	0.53	−1.1	(−1.9, −0.2)	0.02	−0.1	(−0.3, 0.1)	0.43
smoking status			0.13			**0.0006**			0.56			**0.005**
never	0	(reference)		0	(reference)		0	(reference)		0	(reference)	
current	−19.8	(−47.5, 7.9)		**29.2**	**(7.5, 50.9)**		−5.5	(−16.5, 5.5)		−**3.9**	**(**−**6.3**, −**1.5)**	
former	9.0	(−12.2, 30.3)		−6.7	(−23.4, 10.0)		0.1	(−8.4, 8.6)		−2.4	(−4.6, −0.3)	
alcohol consumption			0.24			0.55			0.10			0.15
never	−44.9	(−106.3, 16.6)		16.4	(−33.7, 66.4)		26.1	(−1.2, 53.4)		2.4	(−1.8, 6.7)	
max. 1x/month	0	(reference)		0	(reference)		0	(reference)		0	(reference)	
2–4x/month	−4.0	(−32.7, 24.6)		9.5	(−13.2, 32.2)		−3.4	(−15.1, 8.3)		−2.1	(−5.8, 1.7)	
2–3x/week	−13.3	(−44.1, 17.5)		13.1	(−11.9, 38.0)		0.9	(−10.5, 12.3)		−0.7	(−4.7, 3.3)	
≥4x/week	−32.4	(−66.3, 1.6)		23.2	(−3.8, 50.2)		10.3	(−4.0, 24.5)		−1.1	(−5, 2.8)	
university entrance qualification (yes vs. no)	**35.9**	**(13.0, 58.8)**	**0.002**	−**31.7**	**(**−**49.9**, −**13.4)**	**0.0008**	−4.7	(−13.0, 3.5)	0.26	0.5	(−1.7, 2.7)	0.66
employment status			**<0.0001**			**0.0001**			0.44			0.63
full time	0	(reference)		0	(reference)		0	(reference)		0	(reference)	
part time	−3.4	(−29.2, 22.4)		3.5	(−17.1, 24.1)		1.0	(−9.2, 11.3)		−1.1	(−4.0, 1.8)	
not employed	**66.2**	**(34.7, 97.7)**		−**50.7**	**(**−**76.6**, −**24.8)**		−14.1	(−26.3, −1.8)		−1.4	(−4.4, 1.6)	
net household income per month			0.64			0.85			0.17			0.60
<2,500 €	0	(reference)		0	(reference)		0	(reference)		0	(reference)	
2,500–4,000 €	6.8	(−16.7, 30.4)		−6.4	(−23.4, 10.6)		−0.5	(−11.0, 10.1)		0.1	(−2.1, 2.2)	
>4,000 €	11.8	(−18.8, 42.5)		−7.2	(−30.7, 16.3)		−4.8	(−16.3, 6.7)		0.1	(−3.2, 3.5)	
n. a.	−15.6	(−56.0, 24.8)		−7.8	(−36.9, 21.3)		20.4	(−1.5, 42.3)		3.0	(−1.5, 7.5)	
marital status (married, no vs. yes)	21.3	(−3.7, 46.3)	0.09	−12.1	(−32.6, 8.3)	0.24	−6.3	(−15.5, 2.9)	0.18	−2.9	(−5.7, −0.2)	0.04
diabetes mellitus (yes vs. no)	−18.3	(−72.7, 36.1)	0.51	12.3	(−26.6, 51.2)	0.53	5.3	(−13.3, 23.8)	0.58	0.7	(−1.9, 3.4)	0.59
dyslipidaemia (yes vs. no)	−2.6	(−28.1, 22.9)	0.84	1.6	(−19.2, 22.4)	0.88	0.4	(−8.6, 9.4)	0.93	0.6	(−1.2, 2.3)	0.51

Information was derived from self-reports during a personal interview, anthropometric measures were taken by trained personnel

95% CI, 95% confidence interval; BMI, body mass index; min/d, minutes per day; n. a., not available; vs., versus; VV, vigorous to very vigorous; WC, waist circumference.

^*^Results were derived from four different multivariable linear regression analyses with factors potentially related to physical activity included as independent and time spent in the four different activity intensities included as single dependent variable. β-coefficients can be interpreted as absolute change in time in the different activity intensities in minutes per day, referring to a 1-unit increase for continuous variables or to the respective reference category for categorical variables. Model includes sex, age, body mass index (BMI), waist circumference (residually adjusted for BMI), smoking status, alcohol consumption, university entrance qualification, employment status, net household income, marital status, diabetes, dyslipidaemia, and study centre. Activity intensities were determined based on triaxial 24h-accelerometry vector magnitude defining 0–78 cpm as ‘inactivity’, 79–2,690 cpm as ‘low-intensity’, 2,691–6,166 cpm as ‘moderate’, and ≥6,167 cpm as VV activity^27,28^.

^a^Residually adjusted for BMI.

**Bold**: statistically significant when accounting for multiple testing (*p*-value < 0.01).

### Logistic regression analyses

Regarding the multivariable-adjusted associations of investigated factors with the likelihood of meeting the WHO PA recommendation, current compared to never smokers were substantially less likely to meet the criteria (odds ratio, OR; 95% CI: 0.21; 0.08 to 0.53) (Table 4). Sex differences in these associations were not statistically significant after accounting for multiple testing (Supplementary Table S3).

**Table 4:** Multivariable association of physical activity-related factors and fulfilment of WHO physical activity recommendation^*^, total (N = 249).

potential factors	meeting WHO PA recommendation^a^ (yes vs. no)			
	OR	95% CI	p	
sex (men vs. women)	0.95	(0.35, 2.53)	0.91	
age (5 years)	1.09	(0.89, 1.32)	0.41	
BMI, kg/m²	0.96	(0.87, 1.05)	0.36	
WC, cm^b^	0.97	(0.91, 1.04)	0.42	
smoking status				**0.004**
never	1	(reference)		
current	**0.21**	**(0.08, 0.53)**	**0.001**	
former	0.59	(0.30, 1.16)	0.12	
alcohol consumption				0.68
never	0.32	(0.02, 4.72)	0.41	
maximal 1 × /month	1	(reference)		
2–4 × /month	1.51	(0.62, 3.69)	0.36	
2–3 × /week	1.59	(0.62, 4.09)	0.34	
≥ 4 × /week	1.30	(0.44, 3.87)	0.64	
university entrance qualification (yes vs. no)	1.29	(0.66, 2.51)	0.46	
employment status				0.95
full time	1	(reference)		
part time	0.89	(0.38, 2.08)	0.79	
not employed	1.04	(0.38, 2.85)	0.94	
net household income per month				0.85
<2,500 €	1	(reference)		
2,500–4,000 €	1.09	(0.49, 2.43)	0.83	
>4,000 €	0.86	(0.34, 2.17)	0.75	
n. a.	1.67	(0.39, 7.09)	0.49	
marital status (married, no vs. yes)	1.67	(0.77, 3.62)	0.20	
diabetes mellitus (yes vs. no)	0.58	(0.15, 2.28)	0.44	
dyslipidaemia (yes vs. no)	0.96	(0.44, 2.11)	0.93	

Information was derived from self-reports during a personal interview, anthropometric measures were taken by trained personnel

95% CI, 95% confidence interval; BMI, body mass index; n. a., not available; OR, odds ratio; PA, physical activity; vs., versus; WC, waist circumference; WHO, World Health Organization.

^*^Results were derived from a multivariable logistic regression analysis with factors potentially related to physical activity included as independent and fulfilment of the World Health Organization (WHO) physical activity recommendation included as dependent variable. β-coefficients can be interpreted as change in the likelihood (odds ratio, OR) of meeting the WHO recommendation, referring to a 1-unit increase for continuous variables or to the respective reference category for categorical variables. Model includes sex, age, body mass index (BMI), waist circumference (residually adjusted for BMI), smoking status, alcohol consumption, university entrance qualification, employment status, net household income, marital status, diabetes, dyslipidaemia, and study centre.

^a^‘Meeting the WHO PA recommendation’ (‘yes’) was defined as accumulating ≥150 min/week or ≥75 min/week of vigorous activity/week (here: VV activity) (mean weekly estimates: mean min/d per participant multiplied by 7), spent in activity bouts ≥10 minutes, or an equivalent combination of these^1^. For the latter, metabolic equivalents of tasks (METs)/week were calculated, when multiplying mean weekly estimates in moderate and VV activity by 4 and 8 METs, respectively, as described before^29^. Achieving with the sum of both ≥450 METs/week, this was classified as ‘meeting WHO PA recommendation’. Not meeting any of the aforementioned criteria was classified as ‘not meeting WHO recommendation’.

^b^Residually adjusted for BMI.

**Bold**: statistically significant when accounting for multiple testing (*p*-value <0.01).

### Sensitivity analyses

When repeating the analyses using the 60-minutes instead of 120-minutes accelerometer non-wear time algorithm, results were quite similar to the main analyses, although the association of age, BMI, and employment status with time in low-intensity activity were no longer statistically significant in these analyses (data not shown).

When evaluating the uniaxial accelerometry data, the association of age with time in light and VV activity, of BMI and smoking status with time in light activity, and of university entrance qualification with sedentary time were not statistically significant anymore compared to the equivalent triaxial-derived activity parameters (Supplementary Table S4).

Results for time in inactivity for persons being ‘retired’ and ‘not employed due to other reasons’ were similar as findings for the joint group of not employed persons (retired, yes vs. no: 68.0 min/d; 36.7, 99.3; not employed due to other reasons, yes vs. no: 65.0 min/d; 17.4, 112.6).

## Discussion

The present analyses aimed to identify factors related to time in different PA intensities using multiday 24h-accelerometry in a population-based sample. We found that higher age and current compared to never smoking were associated with more time spent in low-intensity and less time spent in VV activity, while higher BMI was related to less time spent in low-intensity activity. Furthermore, having versus not having a university entrance qualification and no versus full time employment were both associated with more time spent in inactivity and less time in low-intensity activity. Finally, current compared to never smoking was associated with a lower likelihood of meeting the WHO PA recommendation. These mutually adjusted associations were further independent of sex, WC, alcohol consumption, net household income, marital status, diabetes mellitus, dyslipidaemia, and study centre, respectively. The identified factors should be taken into account when planning public health measures aiming to increase PA.

Higher age was related to less time in VV activity and more time in low-intensity activity. These findings are consistent with previous findings from cross-sectional and longitudinal studies, including 24h-accelerometry-based data^29,32–34^. A changing engagement in activity intensities with age is plausible due to an age-related decline in cardiorespiratory and musculoskeletal fitness, thus, limiting resources to perform highly intense PA^35,36^. Furthermore, psychosocial barriers, like a decrease in social support for PA or feeling ‘too old’ for high intensity PA may contribute to the observed decline in VV activity and the simultaneous increase in low-intensity activity with higher age^37^. Although not modifiable, age as a PA-related factor seems to be relevant for activity intensity on the population level. Interestingly, age was not related to time in inactivity, indicating that although higher intense activity may decrease with age, 24h-accelerometry-based inactivity time may not vary significantly.

Additionally, higher BMI was associated with less time spent in low-intensity activity, which is in line with previous findings for accelerometry-based time in light activity^38^. This effect can probably partly be explained by the fact that BMI is strongly correlated with body weight^39,40^ and thus, that persons with higher BMI may more likely be less active (in terms of acceleration) due to the higher effort of movements; in turn, the activity-related energy expenditure does not compulsorily differ from persons with lower BMI^41^. Further, higher BMI is related to an impaired cardiopulmonary fitness that may limit the capacity of being active^42^, and to psychological traits, like a decreased self-efficacy, self-confidence, and perceived fitness that, in turn, are inversely related to PA^9,13^. Interestingly, although reported previously^3,43–45^, we did not find an association of BMI with other activity intensities, but there was a non-significant trend for a positive association with inactivity time. Although the effect size was small for a 1-unit increase in BMI, considering larger changes in this modifiable factor, our analyses indicate an epidemiologically relevant impact of BMI on time in low-intensity activity.

Further, compared to never smokers, current smokers spent less time in VV, while spending almost half an hour more time in low-intensity activity. These observations are plausible, given the negative impact of smoking on cardiopulmonary and musculoskeletal functioning and fitness, energy and oxygen metabolism and supply^46,47^. All of those result in a perceived higher effort, decreased capability, and earlier fatigue, when being active at higher intensity^46,47^, and may, thus, deter smokers from higher intensity activity. Additionally, smoking is often related to a generally less health-conscious lifestyle, including lower sports and exercise engagement^48^, i.e., behaviours of higher intensity. Finally, smokers tend to have a higher discount rate than never smokers, i.e. a higher behavioural impatience and devaluation of delayed outcomes, indicating that they favour an immediate reward (ease of low-intensity activity) even when it is less advantageous above a larger reward being beneficial at a later time (better health)^49,50^, making a preference for low over higher intensity activities reasonable. A previous longitudinal study showed that smoking versus non-smoking is associated with a lower likelihood of being persistently moderately or vigorously active over a 10-years period^32^. Thus, one may conclude that smoking has a long-term effect on intensity of individual PA. This assumption is supported by our observation that former compared to never smokers tended to spent less time in VV activity; however, this association was not statistically significant.

Systematic analyses of reviews and meta-analyses suggest that socio-economic indicators are associated with PA^6,34,51^. We found two socio-economic factors to be related to time spent in inactivity. Firstly, having a university entrance qualification versus no such qualification was associated with more than half an hour more inactivity time, coming at the expense of time in low-intensity activity. This may be surprising, given most previous studies showing higher education to be related to higher self-reported PA levels^6,34,51^. Persons with versus without a university entrance qualification may engage in more targeted activities, like sports, due to more financial resources and a better knowledge of PA benefits. However, on the other hand, they may more likely have less PA-demanding jobs, e.g., white-collar work, while low occupational PA-levels, in turn, are related to low leisure-time PA levels^52^. Further, we did not rely on self-reported activities, as in most previous studies, but used 24h-accelerometry and, thus, continuously measured overall movement across the day. These reasons may explain our findings of a shift from active time at low intensity to more inactivity time when having versus not having a university entrance qualification.

Similarly, no versus full time employment was related to more than one hour more time in inactivity, with this plus coming at the expense of time spent in low-intensity activity. These associations were the strongest observed in the present analyses and are in line with previous studies on self-reported PA^34,51^. A shift from low-intensity to time in inactivity is plausible for unemployment versus full time employment, as it can partly be explained by a lack of transportation (way to work) or occupational PA that may account for a large proportion of habitual PA, especially in manual, blue-collar work^52^. Furthermore, not being employed is associated with the risk of social deprivation, lacking interest in PA, as well as with poorer health and well-being, being, in turn, all related to lower PA engagement^13,33,53^. Therefore, populations with high levels of unemployment, but also higher education and sedentary jobs may represent targets for PA promotion strategies.

Our linear regression analyses include any time of PA spent in the respective PA parameter. The WHO in their definition recommends that these activities should make up at least 150 min/week of moderate or 75 min/week of VV activity spent in bouts of at least 10 minutes. When applying these criteria, we found that current compared to never smoking was associated with a decreased likelihood of meeting the WHO recommendation by 79%. Due to the relevant impact (mean difference in time in activity intensities and OR) and consistency across main and sensitivity analyses, smoking is considered to be an important public health-related lifestyle risk factor associated with PA. In their 2018 edition of PA guidelines for adults, the U.S. Department of Health and Human Services removed the 10-min bouts requirement^54^. In our sample, all participants fulfilled the PA recommendations when based on these US guidelines.

In our analyses, there was a tendency for a sex difference in the associations of WC and smoking with moderate activity and with the likelihood of meeting the WHO PA recommendation, as well as of net household income with inactivity time. However, when accounting for multiple testing, these differences were not statistically significant. Further studies with larger sample sizes are warranted to assess whether there are true differences between sexes.

Most previous studies used uniaxial accelerometry during waking to assess PA^16–19^, whereas newer cohorts like the NAKO or UK Biobank use triaxial 24h-accelerometry^20–22^. While the advantage of triaxial outputs is debated^55^, previous studies did not find substantial differences between uni- and triaxial measures regarding their association with energy expenditure, rather, there was a trend for slightly stronger associations and a more precise PA assessment based on triaxial measures^56–59^. In separate previous analyses, we also did not find significant differences in the association of uni- and triaxial outputs with the activity-related energy expenditure measured by doubly-labelled water. In our present study, we, in line with new epidemiologic studies, rely on triaxial 24h-accelerometry, expecting that this allows a more comprehensive PA assessment, while accepting limited comparability of our findings.

A strength of our study was the focus on multiday triaxial 24h-accelerometry to assess PA in a population-based observational study, thus allowing for a more comprehensive and objective PA assessment than in previous studies using self-reported PA or uniaxial accelerometry during waking only^18,60^. The multicentric study design and application of standard protocols allowed collecting a broad spectrum of participants’ characteristics across Germany with identical methods, thus permitting joint analyses of data. We found no substantial differences between included and excluded participants. Nevertheless, the sample was limited to adults and a selection bias cannot be ruled out. It is well known that participants in epidemiologic studies tend to be more health-conscious than the general population^61^. Findings on PA-related factors may be different in other populations (e.g., diseased populations or other age ranges) and further studies are warranted to investigate PA-related factors in diverse populations. Although participants were asked to pursue their daily routine, study participation and use of accelerometry may affect PA behaviour. However, in a previous study, we did not find any indications of behavioural bias regarding PA when using multiday 24h-accelerometry^27^. Except for anthropometry, factors investigated were self-reported; therefore, social desirability and reporting bias cannot be ruled out. However, if any, we assume such effects to be randomly distributed across PA levels and, thus, to be negligible. Likewise, although we adjusted for a number of variables, residual confounding cannot be ruled out. A further limitation may be that accelerometer data does not entail the domain of PA, such as leisure-time or occupational PA. However, we considered total duration and intensity of PA over 24 h to be the most relevant factor in terms of affecting health. For activity intensity determination, we used a modified version of the ‘Freedson Adult VM3 (2011)’ algorithm, but we were not able to separate sedentary behaviour or sleeping times, which are, however, of epidemiologic interest and are encompassed in ‘time in inactivity’^62–64^. Additionally, since we had no information on accelerometer non-wear times and wake-sleep-rhythms, we used a conservative approach assuming the waking period between 6 AM and 10 PM not allowing for 120 non-wear minutes during this period^26^. However, this waking period may not hold true for all participants, and sleeping during this period may have caused unnecessary exclusion of participants due to falsely detected non-wear time based on accelerometry. If any, this may have limited the power of our analyses, but findings should not have been substantially affected. Our analyses may further include non-wear time periods <120 minutes, which may false-positively be assigned to time in inactivity, when participants were physically active while not wearing the accelerometer^26^. However, results did not substantially change when we used a non-wear time algorithm requiring periods >60 instead of >120 minutes. Compared to previous studies using uniaxial accelerometry during waking, time in moderate and VV activity, and, thus, the proportion of participants meeting the WHO recommendation were slightly higher in our population^29,60^. However, we used triaxial-derived and, thus, other cut points to determine PA intensities than previous studies, and triaxial 24h-accelerometry, making a more comprehensive PA assessment and higher PA levels plausible. This was already observed in our previous study using the same approach^27^. However, we do not expect findings on PA-related factors to be substantially affected by slightly higher group PA levels. It is important to note that our study sample was relatively small and analyses were cross-sectional, thus precluding causal inference, and prospective studies are warranted to further investigate PA-related factors. The comprehensive study protocol and large sample size of the NAKO will provide data for repeating our analyses on a large scale as well as for sound longitudinal analyses on a wide range of determinants and correlates of 24h-accelerometry-based PA^20,21,25^. Finally, it should be noted that non-significant results in our analyses may not inevitably indicate the absence of a true association, but may also be due to the limited sample size.

In conclusion, using multiday 24h-accelerometry to assess habitual PA in a population-based study, our results suggest that the assessed biological, behavioural, socio-economic and socio-cultural factors show distinct associations with 24h-accelerometry-based time in activity intensities. Our findings underpin the relevance of times in different intensities for individual habitual PA, being comprehensively assessable using 24h-accelerometry. The identified modifiable factors may represent targets for public health measures of PA promotion strategies, while the observed unmodifiable PA-related factors allow identification of target subgroups for such programs.

## Supplementary information

Supplementary Tables 1 to 4.

## Data Availability

The data that support the findings of this study are available from the NAKO but restrictions apply to the availability of these data, which were used under license for the current study, and so are not publicly available. Data are however available from the authors upon reasonable request and with permission of the NAKO.
