# Supplementary material for: Factors associated with habitual time spent in different physical activity intensities using multiday accelerometry
Source: Sci Rep. 2020 Jan 21;10:774. doi: 10.1038/s41598-020-57648-w (PMC6972881; doi:10.1038/s41598-020-57648-w)
Supplement: Supplementary file 1 — Supplementary Tables 1 to 4. [file 41598_2020_57648_MOESM1_ESM.pdf]

Factors associated with habitual time spent in different physical activity intensities using multiday accelerometry

Lina Jaeschke[1*], Astrid Steinbrecher[1], Heiner Boeing[2], Sylvia Gastell[2], Wolfgang Ahrens[3,4], Klaus Berger[5], Hermann Brenner[6], Nina Ebert[7], Beate Fischer[8], Karin Halina Greiser[9], Wolfgang Hoffmann[10], Karl-Heinz Jöckel[11], Rudolf Kaaks[9], Thomas Keil[12], Yvonne Kemmling[13], Alexander Kluttig[14], Lilian Krist[12], Michael Leitzmann[8], Wolfgang Lieb[15], Jakob Linseisen[16,17], Markus Löffler[18], Karin B. Michels[19], Nadia Obi[20], Annette Peters[21], Sabine Schipf[22], Börge Schmidt[11], Melanie Zinkhan[14], Tobias Pischon[1,23,24,25]

[1] Molecular Epidemiology Group, Max Delbrück Center for Molecular Medicine in the Helmholtz Association (MDC), Berlin, Germany

[2] Division of Epidemiology, German Institute of Human Nutrition Potsdam-Rehbruecke, Potsdam-Rehbruecke, Germany

[3] Leibniz Institute for Prevention Research and Epidemiology – BIPS, Bremen, Germany

[4] Institute of Statistics, Faculty of Mathematics and Computer Science, University of Bremen, Bremen, Germany

[5] Institute of Epidemiology and Social Medicine, University of Münster, Münster, Germany

[6] Division of Clinical Epidemiology and Aging Research, German Cancer Research Center, INF 581, Heidelberg, Germany

[7] German Diabetes Center (DDZ), Leibniz Center for Diabetes Research, Heinrich Heine University, Institute for Biometrics and Epidemiology, Düsseldorf, Germany

[8] Department of Epidemiology and Preventive Medicine, University of Regensburg, Regensburg, Germany

[9] German Cancer Research Center (DKFZ), Heidelberg, Germany

[10] Section Epidemiology of Health Care and Community Health, Institute for Community Medicine, University Medicine Greifswald, Greifswald, Germany
[11] Institute for Medical Informatics, Biometry and Epidemiology (IMIBE), University Hospital Essen, University of Duisburg-Essen, Essen, Germany
[12] Institute for Social Medicine, Epidemiology and Health Economics, Charité – Universitätsmedizin Berlin, Berlin, Germany
[13] Department of Epidemiology, Helmholtz-Centre for Infection Research, Braunschweig, Germany
[14] Institute of Medical Epidemiology, Biometry and Informatics, Martin-Luther-University, Halle (Saale), Germany
[15] Institute of Epidemiology, Kiel University, Kiel, Germany
[16] Chair of Epidemiology, LMU Munich at UNIKA-T, Augsburg, Germany
[17] Helmholtz Zentrum München, IRG Clinical Epidemiology, Neuherberg, Germany
[18] Institute for Medical Informatics, Statistics and Epidemiology, Leipzig, Germany
[19] Institute for Prevention and Cancer Epidemiology, Faculty of Medicine and Medical Center, University of Freiburg, Freiburg, Germany
[20] Institute for Medical Biometry and Epidemiology, University Medical Center Hamburg-Eppendorf, Hamburg, Germany
[21] Institute of Epidemiology, Helmholtz Zentrum München - German Center for Health and Environment, Neuherberg, Germany
[22] Institute for Community Medicine, University Medicine Greifswald, Greifswald, Germany
[23] Charité – Universitätsmedizin Berlin, Berlin, Germany
[24] German Center for Cardiovascular Research (DZHK), partner site Berlin, Berlin, Germany
[25] MDC/BIH Biobank, Max Delbrück Center for Molecular Medicine and Berlin Institute of Health, Berlin, Germany

***Corresponding author**

Lina Jaeschke, Dr. PH, MPH

Molecular Epidemiology Group, Max Delbrück Center for Molecular Medicine in the Helmholtz Association (MDC), Robert-Roessle-Strasse 10, 13125 Berlin, Germany.

Tel. +4930 9406-4597, Fax +4930 9406-4576; e-mail: lina.jaeschke@mdc-berlin.de

Text count: 4,527 (excl. title page, abstract, references, figures, and tables); abstract, 200

Supplementary Table S1. Participants of pretest 2 of the German National Cohort (NAKO Gesundheitsstudie) per study centre

	total		men		women	
	n	%	n	%	n	%
all study centres	249	100.0	117	100.0	132	100.0
Augsburg	21	8.4	7	6.0	14	10.6
Berlin-Center	23	9.2	9	7.7	14	10.6
Berlin-North	13	5.2	4	3.4	9	6.8
Berlin-South/Brandenburg	21	8.4	9	7.7	12	9.1
Hannover/Braunschweig	15	6.0	9	7.7	6	4.5
Bremen	22	8.8	8	6.8	14	10.6
Düsseldorf	13	5.2	7	6.0	6	4.5
Freiburg	7	2.8	3	2.6	4	3.0
Halle	19	7.6	12	10.3	7	5.3
Hamburg	13	5.2	10	8.5	3	2.3
Heidelberg	23	9.2	8	6.8	15	11.4
Kiel	10	4.0	6	5.1	4	3.0
Münster	6	2.4	3	2.6	3	2.3
Neubrandenburg	25	10.0	15	12.8	10	7.6
Regensburg	14	5.6	5	4.3	9	6.8
Saarbrücken	4	1.6	2	1.7	2	1.5

Supplementary Table S2. Multivariable association of physical activity-related factors and time in different activity intensities[1], sex-stratified

potential factors	**time in inactivity**, min/d						**test for sex differences**[a]	**time in low-intensity activity**, min/d						**test for sex differences**[a]	**time in moderate activity**, min/d						**test for sex differences**[a]	**time in VV activity**, min/d						**test for sex differences**[a]
	**men** (n = 117)			**women** (n = 132)				**men** (n = 117)			**women** (n = 132)				**men** (n = 117)			**women** (n = 132)				**men** (n = 117)			**women** (n = 132)			
	**β**	**95% CI**	**p**	**β**	**95% CI**	**p**	**p**	**β**	**95% CI**	**p**	**β**	**95% CI**	**p**	**p**	**β**	**95% CI**	**p**	**β**	**95% CI**	**p**	**p**	**β**	**95% CI**	**p**	**β**	**95% CI**	**p**	**p**
age (5 years)	-3.8	(-15.8, 8.1)	0.52	-11.9	(-20.4, -3.4)	0.007	0.16	5.1	(-3.8, 14.1)	0.26	13.0	(6.5, 19.5)	0.0001	0.17	-0.9	(-5.0, 3.3)	0.68	0.1	(-3.0, 3.1)	0.96	0.27	-0.4	(-1.3, 0.4)	0.31	-1.2	(-2.0, -0.5)	0.002	0.39
BMI, kg/m²	4.8	(-0.4, 9.9)	0.07	4.0	(-0.6, 8.7)	0.09	0.85	-4.3	(-8.3, -0.3)	0.04	-5.1	(-8.8, -1.5)	0.006	0.85	-0.5	(-2.3, 1.3)	0.57	1.3	(-0.5, 3.0)	0.15	0.69	0.0	(-0.4, 0.4)	0.93	-0.2	(-0.7, 0.4)	0.53	0.24
waist circumference, cm[b]	2.0	(-0.9, 4.8)	0.18	2.2	(-0.2, 4.7)	0.08	0.31	0.1	(-2.1, 2.4)	0.89	-2.2	(-4.1, -0.3)	0.03	0.90	-1.7	(-2.9, -0.6)	0.003	-0.2	(-1.4, 0.9)	0.70	0.03	-0.4	(-0.6, -0.1)	0.002	0.2	(-0.2, 0.6)	0.29	0.06
smoking status			0.27			0.09	0.29			0.30			0.0006	0.48			0.42			0.02	0.03			0.10			0.002	0.70
never	0	(reference)		0	(reference)				(reference)			(reference)				(reference)			(reference)				(reference)			(reference)		
current	-40.7	(-92, 10.6)		-12.5	(-45.3, 20.3)			29.0	(-7.9, 65.9)		35.9	(10.1, 61.7)			13.4	(-7.9, 34.7)		-18.3	(-31.6, -5.0)			-1.7	(-4.8, 1.3)		-5.0	(-7.9, -2.2)		
former	-5.3	(-36.6, 26)		25.1	(-4.1, 54.4)			6.6	(-17.2, 30.5)		-20.8	(-45.3, 3.6)			1.6	(-10.8, 14.1)		-2.8	(-13.5, 7.9)			-3.0	(-5.8, -0.2)		-1.5	(-4.1, 1.0)		
alcohol consumption			0.99			0.007	0.11			0.95			0.009	0.05			0.67			0.05	0.60			0.38			0.48	0.36
never	-19.8	(-122.5, 82.8)		-70.3	(-164.5, 23.8)			12.6	(-64.8, 89.9)		22.4	(-50.0, 94.8)			5.2	(-31.6, 42.1)		47.3	(9.8, 84.8)			2.0	(-4.4, 8.4)		0.6	(-4.8, 6.1)		
max. 1x/month	0	(reference)		0	(reference)				(reference)			(reference)				(reference)			(reference)				(reference)			(reference)		
2 - 4x/month	-3.8	(-54.4, 46.7)		-6.8	(-39.7, 26.1)			13.7	(-22.7, 50.1)		12.2	(-15.9, 40.4)			-7.0	(-25.8, 11.7)		-4.3	(-18.3, 9.7)			-2.8	(-7.0, 1.4)		-1.2	(-4.8, 2.5)		
2 - 3x/week	0.6	(-49.3, 50.6)		-27.8	(-69.5, 14.0)			4.4	(-30.5, 39.4)		22.3	(-12.3, 57.0)			-3.8	(-24.1, 16.5)		4.9	(-9.5, 19.4)			-1.3	(-5.3, 2.7)		0.5	(-4.0, 5.1)		
≥4x/week	-7.3	(-63.9, 49.3)		-65.9	(-105.0, -26.8)			3.6	(-38.2, 45.5)		60.5	(27.6, 93.5)			4.9	(-17.2, 27.0)		10.0	(-7.8, 27.8)			-1.2	(-5.2, 2.8)		-4.7	(-11.3, 1.9)		
university entrance qualification (yes vs. no)	38.4	(6.8, 70.0)	0.02	30.4	(-0.6, 61.4)	0.05	0.27	-37.9	(-63.8, -11.9)	0.005	-25.9	(-50.3, -1.5)	0.04	0.20	-2.6	(-14.5, 9.3)	0.66	-3.3	(-13.7, 7.2)	0.54	0.61	2.1	(-0.0, 4.3)	0.05	-1.2	(-4.9, 2.4)	0.50	0.33
employment status			0.06			0.03	0.59			0.19			0.01	0.88			0.03			0.84	0.19			0.39			0.67	0.61
full time	0	(reference)		0	(reference)				(reference)			(reference)				(reference)			(reference)				(reference)			(reference)		
part time	-18.0	(-60.0, 23.9)		-5.4	(-39.1, 28.2)			2.6	(-29.2, 34.4)		6.0	(-21.1, 33.1)			15.9	(-4.2, 36.0)		0.1	(-13.2, 13.5)			-0.5	(-5.1, 4.1)		-0.7	(-3.9, 2.5)		
not employed	63.4	(6.1, 120.7)		46.4	(3.8, 88.9)			-37.4	(-78.2, 3.5)		-41.0	(-75.5, -6.4)			-23.6	(-46.4, -0.7)		-3.8	(-21.1, 13.4)			-2.4	(-6.1, 1.2)		-1.6	(-5.2, 2.0)		
net household income per month			0.10			0.38	0.02			0.148			0.59	0.10			0.003			0.21	0.05			0.002			0.39	0.61
<2,500 €	0	(reference)		0	(reference)				(reference)			(reference)				(reference)			(reference)				(reference)			(reference)		
2,500-4,000 €	30.1	(-6.9, 67.2)		-17.4	(-48.6, 13.9)			-21.7	(-46.6, 3.2)		8.9	(-16.1, 34.0)			-7.7	(-24.4, 9.0)		7.5	(-5.4, 20.3)			-0.7	(-4.1, 2.7)		1.0	(-1.7, 3.7)		
>4,000 €	48.8	(9.6, 87.9)		-40.2	(-88.9, 8.4)			-23.6	(-53.5, 6.6)		25.8	(-12.6, 64.1)			-24.0	(-40.4, -7.6)		12.7	(-3.3, 28.6)			-1.2	(-4.5, 2.1)		1.8	(-2.7, 6.4)		
n. a.	20.9	(-32.9, 74.7)		-33.4	(-91.9, 25.0)			-36.0	(-75.4, 3.5)		2.4	(-38.7, 43.5)			9.9	(-13.6, 33.3)		27.3	(-1.1, 55.7)			5.2	(1.9, 8.5)		3.8	(-1.3, 9.0)		
marital status (married, no vs. yes)	26.6	(-15.5, 68.6)	0.21	1.0	(-31.3, 33.3)	0.95	0.87	-8.4	(-41.6, 24.7)	0.61	-3.1	(-29.6, 23.3)	0.82	0.79	-13.9	(-26.7, -1.2)	0.03	3.1	(-10.0, 16.1)	0.64	0.40	-4.2	(-7.2, -1.2)	0.006	-1.0	(-4.1, 2.1)	0.52	0.26
diabetes mellitus (yes vs. no)	-41.8	(-108.2, 24.6)	0.21	33.8	(-34.7, 102.4)	0.33	0.21	27.6	(-16.9, 72.1)	0.22	-20.8	(-73.0, 31.4)	0.43	0.19	15.9	(-10.0, 41.8)	0.22	-11.3	(-34.1, 11.5)	0.33	0.33	-1.8	(-6.1, 2.6)	0.42	-1.8	(-10.3, 6.7)	0.68	0.94
dyslipidaemia (yes vs. no)	4.1	(-33.7, 42.0)	0.83	-1.7	(-33.8, 30.3)	0.91	0.63	-3.4	(-34.8, 28.1)	0.83	0.7	(-25.4, 26.8)	0.96	0.52	-0.5	(-12.3, 11.3)	0.94	0.1	(-12.3, 12.5)	0.99	0.96	-0.3	(-3.1, 2.5)	0.86	1.0	(-1.3, 3.2)	0.39	0.78

information was derived from self-reports during a personal interview, anthropometric measures were taken by trained personnel

95% CI, 95% confidence interval; BMI, body mass index; min/d, minutes per day; n. a., not available; vs., versus; VV, vigorous-to-very-vigorous; WC, waist circumference

[1]Results were derived from four different multivariable linear regression analyses with factors potentially related to physical activity included as independent and time spent in the four different activity intensities included as single dependent variable. β-coefficients can be interpreted as absolute change in time in the different activity intensities in minutes per day, referring to a 1-unit increase for continuous variables or to the respective reference category for categorical variables. Model includes sex, age, body mass index (BMI), waist circumference (residually adjusted for BMI), smoking status, alcohol consumption, university entrance qualification, employment status, net household income, marital status, diabetes, dyslipidaemia, and study centre. Activity intensities were determined based on triaxial 24h-accelerometry vector magnitude defining 0-78 cpm as ‘inactivity’, 79-2,690 cpm as ‘low-intensity’, 2,691-6,166 cpm as ‘moderate’, and ≥6,167 cpm as VV activity [27, 28].

[a]continuous variables, normally distributed: t-test; continuous variables, not normally distributed (absolute and relative proportion in vigorous-to-very-vigorous activity, bout parameters): Mann-Whitney U test; discrete variables: Chi-Square test

[b]residually adjusted for BMI

Supplementary Table S3. Multivariable association of physical activity-related factors and fulfilment of World Health Organization physical activity recommendation[1], sex-stratified

potential factors	meeting WHO PA recommendation[a] (yes vs. no) men (n = 117) OR	95% CI	p	women (n = 132) OR	95% CI	p	test for sex differences[b] p
age (5 years)	1.73	(0.99, 3.01)	0.05	0.75	(0.56, 1.02)	0.07	0.09
BMI, kg/m²	0.76	(0.56, 1.03)	0.08	1.09	(0.94, 1.27)	0.27	0.30
WC, cm[c]	0.79	(0.65, 0.97)	0.02	1.11	(1.01, 1.23)	0.03	0.03
smoking status							
never	1	(reference)	0.03	1	(reference)	0.03	0.03
current	0.08	(0.01, 0.83)		0.22	(0.05, 0.93)		
former	0.05	(0.01, 0.47)		1.67	(0.59, 4.72)		
alcohol consumption							0.93
never	n. a.[d]	n. a.[d]	0.99	1.83	(0.08, 41.98)	0.98	
max. 1x/month	1	(reference)		1	(reference)		
2 - 4x/month	0.79	(0.06, 9.67)		1.43	(0.41, 5.05)		
2 - 3x/week	0.81	(0.06, 11.26)		1.20	(0.32, 4.54)		
≥4x/week	0.50	(0.03, 7.53)		1.02	(0.19, 5.46)		
university entrance qualification (yes vs. no)	6.06	(1.11, 33.13)	0.04	0.76	(0.27, 2.11)	0.60	0.22
employment status							0.20
full time	1	(reference)	0.61	1	(reference)	1.00	
part time	0.44	(0.02, 9.28)		1.00	(0.30, 3.33)		
not employed	2.52	(0.15, 43.14)		1.00	(0.20, 5.03)		
net household income per month							0.64
<2,500 €	1	(reference)	0.62	1	(reference)	0.28	
2,500-4,000 €	2.55	(0.32, 20.28)		0.80	(0.25, 2.58)		
>4,000 €	0.53	(0.07, 4.34)		0.24	(0.05, 1.28)		
n. a.	1.27	n. a.[d]		1.38	(0.20, 9.60)		
marital status (married, no vs. yes)	3.17	(0.50, 19.91)	0.22	1.95	(0.58, 6.54)	0.28	0.34
diabetes mellitus (yes vs. no)	0.01	(<0.001, 0.90)	0.05	1.47	(0.21, 10.18)	0.70	0.12
dyslipidaemia (yes vs. no)	3.93	(0.60, 25.6)	0.15	1.11	(0.33, 3.69)	0.87	0.53

information was derived from self-reports during a personal interview, anthropometric measures were taken by trained personn
95% CI, 95% confidence interval; BMI, body mass index; min/d, minutes per day; n. a., not available; OR, odds ratio; PA, physical activity; vs., versus; WC, waist circumference; WHO, World Health Organization

[1]Results were derived from a multivariable logistic regression analysis with factors potentially related to physical activity included as independent and fulfilment of the World Health Organization (WHO) physical activity recommendation included as dependent variable. β-coefficients can be interpreted as change in the likelihood (odds ratio, OR) of meeting the WHO recommendation, referring to a 1-unit increase for continuous variables or to the respective reference category for categorical variables. Model includes sex, age, BMI, waist circumference (residually adjusted for BMI), smoking status, alcohol consumption, university entrance qualification, employment status, net household income, marital status, diabetes, dyslipidaemia, and study centre.

[a]'meeting the WHO PA recommendation' ('yes') was defined as accumulating ≥150 min/week or ≥75 min/week of vigorous activity/week (here: VV activity) (mean weekly estimates: mean min/d per participant multiplied by 7), spent in activity bouts ≥10 minutes, or an equivalent combination of these [1]. For the latter metabolic equivalents of tasks (METs)/week were calculated, when multiplying mean weekly estimates in moderate and VV activity by 4 and 8 METs, respectively, as described before [29]. Achieving with the sum of both ≥450 METs/week, this was classified as 'meeting WHO PA recommendation'. Not meeting any of the aforementioned criteria was classified as 'not meeting WHO recommendation'.

[b]continuous variables, normally distributed: t-test; continuous variables, not normally distributed (absolute and relative proportion in vigorous to very vigorous activity, bout parameters): Mann-Whitney U test; discrete variables: Chi-Square test

[c]residually adjusted for BMI

[d]counts per cell too small to calculate meaningful OR and 95% CI

Supplementary Table S4: Multivariable association of physical activity-related factors and time in sedentary behaviour and different activity intensities, uniaxial analyses[1], total (N=249)

**potential factors**	**time in sedentary behaviour** min/d			**time in light activity,** min/d			**time in moderate activity,** min/d			**time in VV activity,** min/d		
	**β**	**95% CI**	**p**	**β**	**95% CI**	**p**	**β**	**95% CI**	**p**	**β**	**95% CI**	**p**
sex (men vs. women)	6.9	(-17.1, 30.9)	0.57	-11.9	(-31.0, 7.3)	0.22	4.9	(-4.1, 13.9)	0.28	1.0	(-3.1, 5.1)	0.64
age (5 years)	-0.3	(-5.9, 5.2)	0.91	1.8	(-3.1, 6.6)	0.48	-0.9	(-2.5, 0.6)	0.25	-0.7	(-1.3, -0.1)	0.01
BMI, kg/m²	0.08	(-2.8, 2.9)	0.96	0.0	(-2.2, 2.3)	0.97	0.1	(-0.9, 1.1)	0.84	-0.2	(-0.6, 0.1)	0.13
WC, cm[a]	1.2	(-0.4, 2.8)	0.13	-0.7	(-1.9, 0.5)	0.23	-0.5	(-1.1, 0.1)	0.11	-0.1	(-0.3, 0.1)	0.46
smoking status			0.75			0.19			0.02			**0.003**
never	0	(reference)		0	(reference)		0	(reference)		0	(reference)	
current	-3.8	(-27.1, 19.4)		15.5	(-4.4, 35.3)		-9.3	(-16.6, -2.0)		**-3.9**	**(-6.1, -1.6)**	
former	4.3	(-13.3, 21.9)		-1.9	(-16.4, 12.6)		-0.7	(-6.7, 5.4)		-2.5	(-4.6, -0.5)	
alcohol consumption			0.17			0.27			0.23			0.45
never	-50.9	(-101.6, -0.2)		31.6	(-4.3, 67.5)		17.4	(-5.7, 40.4)		0.5	(-3.8, 4.7)	
max. 1x/month	0	(reference)		0	(reference)		0	(reference)		0	(reference)	
2 - 4x/month	-4.6	(-28.5, 19.4)		3.8	(-16.4, 24.0)		1.9	(-5.4, 9.2)		-2.2	(-5.8, 1.4)	
2 - 3x/week	-6.5	(-30.5, 17.5)		1.9	(-18.3, 22.1)		4.9	(-2.9, 12.8)		-0.8	(-4.7, 3.0)	
≥4x/week	-26.0	(-54.3, 2.4)		17.7	(-5.0, 40.3)		9.7	(-0.2, 19.5)		-1.1	(-4.8, 2.6)	
university entrance qualification (yes vs. no)	22.0	(3.7, 40.2)	0.02	**-23.7**	**(-38.8, -8.6)**	**0.002**	1.6	(-4.4, 7.5)	0.60	0.5	(-1.6, 2.6)	0.65
employment status			**<.0001**			**0.005**			0.22			0.57
full time	0	(reference)		0	(reference)		0	(reference)		0	(reference)	
part time	-6.1	(-28.0, 15.8)		6.7	(-12.2, 25.7)		-0.1	(-7.4, 7.2)		-1.1	(-3.8, 1.7)	
not employed	**34.3**	**(9.2, 59.4)**		**-27.0**	**(-48.0, -6.0)**		-6.5	(-14.5, 1.6)		-1.5	(-4.4, 1.3)	
net household income per month			0.22			0.31			0.15			0.76
<2,500 €	0	(reference)		0	(reference)		0	(reference)		0	(reference)	
2,500-4,000 €	5.3	(-14.9, 25.5)		-9.0	(-24.6, 6.7)		3.5	(-3.9, 10.9)		0.0	(-2.1, 2.1)	
>4,000 €	13.8	(-10.2, 37.9)		-14.0	(-33.3, 5.3)		-0.8	(-8.9, 7.3)		-0.1	(-3.2, 3.1)	
n. a.	-28.8	(-66.8, 9.2)		13.5	(-20.3, 47.3)		12.6	(0.5, 24.7)		2.2	(-1.9, 6.2)	
marital status (married, no vs. yes)	21.9	(1.7, 42.2)	0.03	-16.8	(-33.8, 0.2)	0.05	-3.5	(-10.3, 3.3)	0.31	-2.7	(-5.3, -0.1)	0.04
diabetes mellitus (yes vs. no)	-12.4	(-56.5, 31.6)	0.58	7.7	(-25.5, 40.8)	0.65	5.1	(-8.2, 18.4)	0.45	0.7	(-1.9, 3.2)	0.62
dyslipidaemia (yes vs. no)	-9.4	(-29.3, 10.5)	0.35	8.8	(-8.0, 25.7)	0.30	0.1	(-6.1, 6.3)	0.98	0.6	(-1.1, 2.2)	0.50

information was derived from self-reports during a personal interview, anthropometric measures were taken by trained personnel

95% CI, 95% confidence interval; BMI, body mass index; min/d, minutes per day; n. a., not available; vs., versus; VV, vigorous to very vigorous; WC, waist circumference

[1]Results were derived from four different multivariable linear regression analyses with factors potentially related to physical activity included as independent and time spent in sedentary behaviour and in light, moderate, and vigorous-to-very-vigorous included as single dependent variable. β-coefficients can be interpreted as absolute change in time in the different activity measures in minutes per day, referring to a 1-unit increase for continuous variables or to the respective reference category for categorical variables. Model includes sex, age, body mass index (BMI), waist circumference (residually adjusted for BMI), smoking status, alcohol consumption, university entrance qualification, employment status, net household income, marital status, diabetes, dyslipidaemia, and study centre. Activity intensities were determined based on uniaxial 24h-accelerometry defining 0-99 cpm measured by the vertical axis as 'sedentary', 100-1,951 cpm as 'light', 1,952-5724 cpm as 'moderate', and ≥5,725 cpm as VV activity [31].

[a]residually adjusted for BMI

bold: statistically significant when accounting for multiple testing (p-value <0.01)
